# The rhizospheric bacterial diversity of *Fritillaria taipaiensis* under single planting pattern over five years

**DOI:** 10.1038/s41598-022-26810-x

**Published:** 2022-12-29

**Authors:** You Zhou, Maojun Mu, Min Yang, Xiaohong Yang, Hua Zhang, Dongqin Guo, Nong Zhou

**Affiliations:** 1 College of Biology and Food Engineering, Chongqing Three Gorges University, Chongqing, 404120 China; 2 College of Pharmacy and Chemistry, Dali University, Dali, 671000 China; 3 College of Horticulture and Landscape Architecture, Southwest University, Chongqing, 400715 China; 4 Jiangsu Key Laboratory of Chinese Medicine Processing, Engineering Center of State Ministry of Education for Standardization of Chinese Medicine Processing, Nanjing University of Chinese Medicine, Nanjing, 210023 China

**Keywords:** Biochemistry, Ecology, Microbiology

## Abstract

Rhizospheric microorganisms can profoundly influence the nutritional status of soil and the growth of plant. To reveal the change on the bacterial diversity in the rhizosphere of *Fritillaria taipaiensis* under long-term single planting, the bacterial community structure in the rhizospheric soils of *F. taipaiensis* with different cultivation years from 1 to 5 were analyzed. The result showed the Chao1 and the ACE indices of the bacterial community had no significant difference among samples while the Shannon and Simpson indices declined with the cutivation year; the intra group beta diversity of the rhizospheric bacteria increased after a initial decline with the cultivation year; in the sample with 1 year of cultivation, the dominant bacterial genera were mainly the species that can improve the soil nutrient status and promote plant growth while with the increase of cultivation year, the dominant genera in samples then gradually reflected the pathogen accumulation and soil nutrient status deterioration; pH was the most significant factor affected by the bacterial community composition. These results indicated long term continuous cropping changed the bacterial community structure and soil nutritional status in the *F. taipaiensis* rhizospheric soils, which could badly affect its growth.

## Introduction

Chuan BeiMu is a kind of the precious traditional Chinese herbal medicine, its source materials are the bulbs of 5 species in genus *Fritillaria* (*Fritillaria cirrhosa* D. Don, *Fritillaria przewalski*i Maxim., *Fritillaria unibracteata* Hsiao et K. C. Hsia, *Fritillaria delavayi* Franch. and *Fritillaria taipaiensis* P. Y. Li)^1^. The Chuan Beimu medicine contain multiple compounds such as alkaloids, steroids, terpenoids, purine and fatty acid, which can cool down body temperature, relieve coughing and asthma, and resolve phlegm^2–4^. *Fritillaria taipaiensis* P. Y. Li, which has been collected in Chinese Pharmacopoeia as one of the medical material provenances of Chuan BeiMu in 2015^5^, has important economic value. In recent years, the production of *F. taipaiensis* has been popularized in suitable areas^6–8^. However, in the actual production, the seedlings of *F. taipaiensis* may appear the problems such as growth potential reducing^9^, disease accumulation^10^, and quality decline^11^ after 3–4 years’ continuous planting, which badly affected its production and quality. In response to this situation, current researches mainly focus on the fertilization control and the planting technology improvement^12,13^.

Soil contains different and wide variety of microbial communities, and the interaction between soil microorganism and soil factors plays an important role in the process of soil organic matter degradation and soil nutrient cycling^14,15^. Meanwhile, the quality of soil can also be reflected by the soil enzyme activities and the microbial community composition^15,16^. Researches have demonstrated continuous cropping and long-term single plating pattern could affect the diversity and structure of the microorganism population in the planting soil^17–19^, these microorganism and then could directly or indirectly affect the growth of plants by changing the chemical properties of soil^20^. In addition, the pathogenic microorganisms accumulated during the long-term single planting can also hinder the growth of plants^21–24^. The studies on the changes of rhizospheric microbial community structure during the *F. taipaiensis* cultivation have not been deeply progressed at present.

In this study, the population structure compositions, richness and evenness of the bacteria in the rhizospheric soils, in which *F. taipaiensis* was planted for 1–5 years, were compared and analyzed to reveal the change on the bacterial diversity in the rhizosphere of *Fritillaria taipaiensis* under long-term single planting. Moreover, we analyzed the relationship between the soil bio-chemical factors and the rhizospheric bacterial community to explore the interaction between soil environment and soil microorganisms. The result of this study could provide a theoretical basis for the soil management in the sustainable cultivation of *F. taipaiensis*.

## Materials and methods

### Sampling site location

The sampling site is a private planting base located in Lanying Village, Wuxi County, Chongqing, China (31°23′56.11″ N, 109°50′29.93″ E-31°35′26.57″ N, 109°00′11.96″ E; 2 274–2 290 m above sea level), with the annual average temperature of 7.2 °C, and the annual average precipitation of 1 100–1 300 mm, the soil in the study site was classified as mountain yellow–brown soil (fine-loamy, mixed, mesic Aridic Haplustalf)^25^. The field was 20 × 20 m for the *F. taipaiensis* with each cultivation year, The plant and row spacing of *F. taipaiensis* was 15 × 15 cm, and the fields were uncultivated wastelands with the same soil basement condition before *F. taipaiensis* planting. The fertilization of the field was N-P-K 15:15:15, 225 kg hm^−2^ for each year.

### Sample collection

In May, 2017, with the permission of the planting base owner, *F. taipaiensis* rhizospheric soils with different cultivation years from 1 to 5 (Y1: rhizospheric soil with 1 year of cultivation, Y2: rhizospheric soil with 2 years of cultivation, Y3: rhizospheric soil with 3 years of cultivation, Y4: rhizospheric soil with 4 years of cultivation, Y5: rhizospheric soil with 5 years of cultivation) were sampled. 3 sampling plots (3 × 3 m) were randomly selected in the field with each cultivation year. The regolith, impurities and gravels on the surface of fields were removed to expose the roots, and the soils attached on the roots 6 ~ 10 cm below the ground were collected by shaking root method^13^. Soils from the rhizosphere of 5 *F. taipaiensis* plants randomly selected in each sampling plot were collected and then were mixed. Then the soil samples were placed into the aseptic bags in ice box and then were transferred into laboratory after removed the litters and residual cover. The experimental samples were sifted by 2 mm sieve and then were stored in − 80 °C ultra-low temperature freezer after freeze drying to be used in soil total DNA extraction. The bio-chemical properties^26^ of the *F. taipaiensis* rhizospheric soils with different cultivation years were shown in Table 1.

**Table 1:** Chemical properties and enzyme activities of *F. taipaiensis* rhizospheric soils with different cultivation years.

Enzyme activity/Content/pH	Samples				
	Y1	Y2	Y3	Y4	Y5
pH	6.43 c	6.50 c	6.52 c	7.47 a	6.85 b
Enzyme activity/Content	g kg^-1^ 24 h^-1^				
Acid phosphatase	0.739 a	0.588 ab	0.484 b	0.452 b	0.694 a
Alkaline phosphatase	0.139 a	0.112 b	0.103 bc	0.089 c	0.066 d
Urease	5.418 ab	5.673 a	5.246 ab	4.399 c	4.743 bc
Invertase	12.826 c	9.569 d	9.250 d	21.783 b	28.959 a
Protease	0.291 c	0.281 c	0.288 c	0.361 b	0.480 a
Catalase	g kg^-1^ 20 min^-1^				
	0.685 a	0.551 b	0.539 b	0.688 a	0.680 a
Organic matter	g kg^-1^				
	36.049 a	28.836 b	26.135 b	21.434 c	21.334 c
	mg kg^-1^				
Available nitrogen	113.012 b	135.315 a	114.056 b	81.324 c	79.224 c
Available phosphorus	150.478 a	96.490 b	97.089 b	44.728 c	39.425 d
Available potassium	405.865 a	174.810 d	210.419 c	169.305 d	302.991 b

Different lower-case letters indicate significant differences (*p* < 0.05) among the 5 treatments.

### Soil DNA extraction and high throughput sequencing

0.25 g *F. taipaiensis* rhizospheric soil sample was used for DNA extraction by CTAB method^27^. The genomic DNA after diluted to 1 ng μl^-1^ was amplified by PCR via high-fidelity DNA polymerase and Phusion® High-Fidelity PCR Master Mix with GC Buffer (New England Biolabs) by using specific primer 515F (5′-GTGCCACCMGCCGCGGTAA-3′) and 806R (5′-GGACTACHVGGGTWTCTAAT-3′) for the bacterial 16sRNA gene V4 sequence, the amplification condition was pre-denaturation at 98 °C for 2 min, denaturation at 98 °C for 30 s, annealing at 50 °C for 30 s, extending at 72 °C for 1 min, after 30 cycles, and then extending 72 °C again for 5 min to end the PCR reaction. The PCR products were tested by 2% agarose gel electrophoresis and then were extracted by gel extraction kit (Qiagen). The DNA library was constructed by using TruSeq® DNA PCR-Free Sample Preparation Kit (Illumina) and then was sequenced by HiSeq2500 PE250.

### Data analysis

The data of each sample was separated from the off machine data according to the Barcode sequence and PCR amplification primer sequence, and the reads of each sample were assembled by FLASH (V1.2.7, http://ccb.jhu.edu/software/FLASH/) after removed the Barcode sequence and the PCR amplification primer sequence to obtain the Raw date. The Raw date were filtered and spliced to get Clean date refer to the Tags quality control process in QIIME (V1.9.1, http://qiime.org/scripts/split_libraries_fastq.html). And then, the chimeras in the Clean date were eliminated by comparing with UCHIME (Algorithm, http://www.drive5.com/usearch/manual/uchime_algo.html) and database (Unite database, https://unite.ut.ee/) to obtain the Effective tags. The depth of sequencing was 40 000 reads at least in each original library, the operational taxonomic unit (OTU) was analyzed and clustered by using the UPARSE software (version 7.0.1090) based on 97% similarity. According to the result of OTU clustering, the representative sequence of each OTU was annotated by a species by using QIIME (version 1.9.1). The relative abundance was analyzed via Mothur (version.1.44.0) in the SS rRNA database of SILVA (http://arb-silva.de/).

The Mothur software (version.1.44.0) was used to calculate the Goods-coverage, Chao1, ACE, Shannon and Simpson indices. The QIIME software (version 1.9.1) was used to calculate the Unifrac distance and plot the unweighted pair-group method using arithmetic average (UPGMA) cluster tree. The R-software (version 2.15.3) was used to plot the Rank abundance curve. Meanwhile, the relative abundance, the Alpha diversity of OTUs was analyzed to get the information such as the unique species abundance in one sample, the common and the unique OTUs among the different samples and groups by Tukey-test. Non-Metric Multi-Dimensional Scaling (NMDS) analysis, distance-based redundancy analysis (db-RDA) and permutational MANOVA analysis (Adonis) were performed by vegan bag on R-software.

The data were analyzed by single factor (one-way ANOVA) and Duncan for variance analysis and multiple comparison via SPSS 22.0 and Excel 2003 (α = 0.05). Correlation analysis was conducted on SPSS 22.0 using the Pearson method (α = 0.05).

## Results

### Alpha diversity analysis

**Figure 1:**
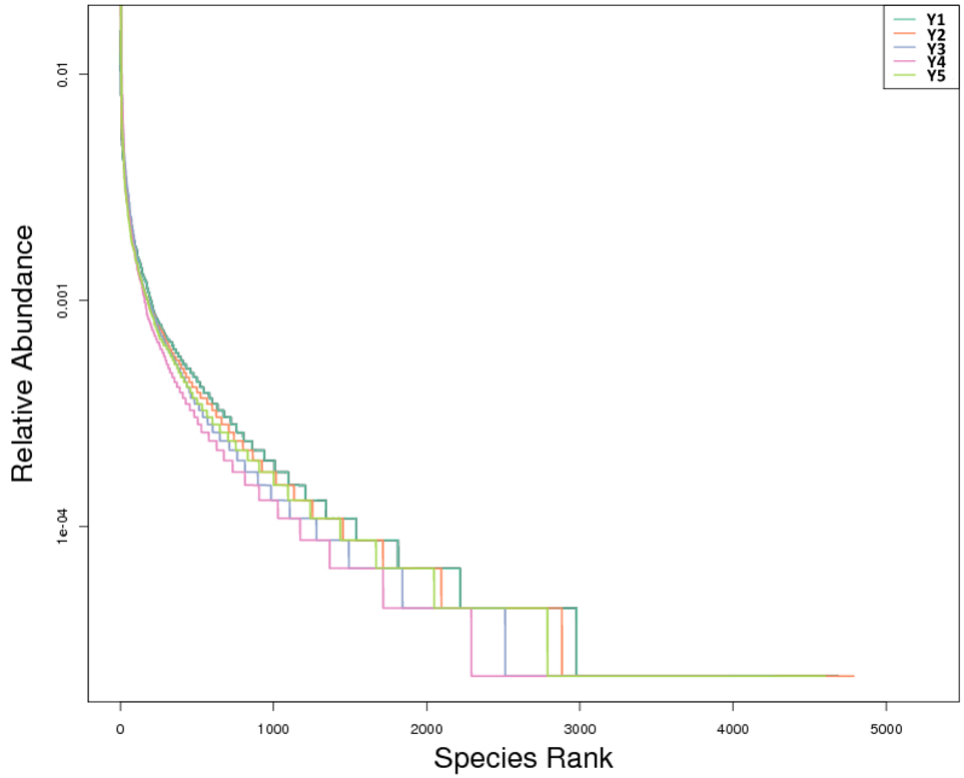
Rank abundance curve. In the horizontal direction, the wider the abscissa span, the higher the species richness; in the vertical direction, the smoother the curve, the higher the species evenness.

The Rank abundance curves (Fig. 1) which were drawn according to the relative abundance of the OTUs showed: the curve of sample Y2 had the maximum abscissa span, which indicated that the *F. taipaiensis* rhizospheric soil with 2 years of cultivation had the maximum bacterial species richness; and the curve of sample Y1 had the softest changing trend in ordinate which indicated the *F. taipaiensis* rhizospheric soil with 1 year of cultivation had the maximum bacterial species evenness; the curve of sample Y4 had the minimum abscissa span and the maxim ordinate changing trend which indicated the *F. taipaiensis* rhizospheric soil with 4 years of cultivation had the minimum bacterial species richness and evenness.

As shown in Table 2: Goods_coverage showed the species coverage detected in each sample was above 97%; there was no significant difference in the Chao1 and ACE indices between the samples, which reflected the species richness of the samples with different cultivation years had no significant change; meanwhile, the Shannon and Simpson indices of the samples showed a trend of declining with the cultivation year, which indicated the reduction of bacterial community diversity.

**Table 2:** Alpha diversity indices.

Sample	Goods_coverage	Chao1	ACE	Shannon	Simpson
Y1	0.978 a	4807.988 a	4849.949 a	10.199 a	0.998 a
Y2	0.976 a	4870.574 a	5021.414 a	9.934 ab	0.996 ab
Y3	0.978 a	4354.877 a	4565.658 a	9.505 b	0.995 b
Y4	0.975 a	4530.162 a	4793.504 a	8.944 c	0.985 c
Y5	0.975 a	4987.040 a	5117.301 a	9.678 b	0.993 b

Goods_coverage: indicator reflects the depth of sequencing, the closer the value is to 1, the more reasonable the sequencing depth, indicating that the sequencing depth has covered all species in the sample; Chao1: provides an estimation of the actual number of OTUs in the sample as an indicator of species richness, the higher value means higher species richness; ACE: an indicator of species richness, the higher value means higher species richness, with a different algorithm to Chao1; Shannon: an index quantifies the entropy of the distribution, which depends on the evenness and richness of the proportional abundances of OTUs in the population, the higher value means higher species diversity; Simpson: an indicator of species diversity, the higher value means higher species diversity with a different algorithm to Shannon; different lower-case letters indicate significant differences (*p* < 0.05) among the 5 treatments.

### Beta diversity analysis of the bacterial communities in the rhizospheric soils

**Figure 2:**
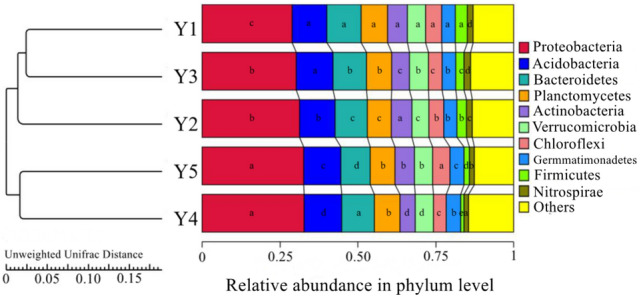
UPGMA dendrogram and relative abundance of main bacterial phyla across sampling over years. Different lower-case letters indicate significant differences (*p* < 0.05) among the six treatments, the same as follow.

As shown in Fig. 2, the bacterial phyla with the top 10 relative abundances in the *F. taipaiensis* rhizospheric soils with different cultivation years were Proteobacteria (34.16–54.82%), Acidobacteria (10.19–18.16%), Bacteroidetes (9.49–13.75%), Verrucomicrobia (4.91%-7.21%), Firmicutes (0.18–6.05%), Gemmatimonadetes (2.87–5.93%), Actinobacteria (1.51–4.55%), Planctomycetes (2.05–3.06%), Nitrospirae (1.01–2.48%) and Chloroflexi (0.96–1.83%). The dominant phylum was Proteobacteria which presented a rising relative abundance from Y1 to Y4 with the cultivation year. And the relative abundance of Firmicutes, Actinobacteria and Chloroflexi showed a downward changing trend from Y1 to Y4, in which the relative abundance of Firmicutes had great variation among samples, declined from 6.05% in Y1 to 0.65% in Y3. Meanwhile, the samples were clustered by unweighted pair group method with arithmetic means (UPGMA). Samples Y4 and Y5 could be grouped together but be separated from the cluster of samples Y1, Y2 and Y3, which revealed that the samples with 4 and 5 years of cultivation had the similar bacterial community structures but were different from the samples with 1, 2, and 3 years of cultivation. The samples Y1, Y2, and Y3 could be grouped in a large cluster, in which the samples with 1 and 3 years of cultivation could be further grouped in a smaller cluster.

The bacterial genera with the top 15 relative abundances in the *F. taipaiensis* rhizospheric soil were *Methylotenera*, *Pseudomonas*, *Lactobacillus*, *Gemmatimonas*, *Ralstonia*, *Bryobacter*, *Sphingobium*, *Flavobacterium*, *Collimonas*, *Sphingomonas*, *Candidatus*, *Rhizomicrobium*, *Pedobacter*, *Bacteroides* and unculturable bacterium RB41 (Table 3). The relative abundances of *Methylotenera*, *Pseudomonas* and *Sphingobium* showed a tendency of ascending with the cultivation year, the relative abundance of *Methylotenera* in Y4 was 16.07 times of that in Y1, and the relative abundance of *Pseudomonas* and *Sphingobium* in Y5 was 5.21 and 4.15 times of that in Y1, respectively. The relative abundances of *Lactobacillus*, *Gemmatimonas*, *Bryobacter* and *Bacteroides* declined with the cultivation year, the relative abundance of *Gemmatimonas*, *Bryobacter* and *Bacteroides* in Y5 declined by 4.67, 2.29, and 99 times compared with that in Y1, respectively, and the relative abundances of *Lactobacillus* in Y4 and Y5 were absent. The relative abundances of *Flavobacterium*, *Collimonas* and *Pedobacter* showed a downward trend after a initial rising, the relative abundance of *Flavobacterium* in Y4 and the relative abundance of *Collimonas* in Y3 was 5.89 and 12 times of that in Y1, respectively. While in Y5, the relative abundances of these 2 genera declined significantly when compared with those in Y3 and Y4, respectively. The relative abundances of *Pedobacter* had no obvious change in Y1, Y2 and Y3 but rose suddenly to a high level in Y4 and then declined obviously in Y5. Meanwhile, the relative abundances of *Candidatus* also had no significant change in Y1, Y2 and Y3 but declined obviously in Y4 and Y5. All the results above indicated that *F. taipaiensis* rhizospheric soils with different cultivation years had significant difference in the dominate bacteria community on the genus level.

**Table 3:** Relative abundance (%) of bacterial genera in the samples with different cultivation years.

Genus	Relative abundance (%)				
	Y1	Y2	Y3	Y4	Y5
*Methylotenera*	0.61 c	2.78 b	2.89 b	8.90 a	3.98 b
*Pseudomonas*	0.92 c	2.13 bc	2.61b	2.18 bc	4.79 a
*Lactobacillus*	2.68 a	1.40 ab	0.78 b	0.00 b	0.00 b
*Gemmatimonas*	2.52 a	0.92 b	0.90 b	0.37 b	0.54 b
*Ralstonia*	1.26 b	1.26 b	1.01 b	2.39 a	1.98 a
*Bryobacter*	2.24 a	2.29 a	2.11 a	0.63 c	0.98 b
*Sphingobium*	0.53 b	1.75 a	1.73 a	2.04 a	2.20 a
*Flavobacterium*	0.35 c	0.32 c	1.13 b	2.06 a	0.62 bc
*Collimonas*	0.17 b	0.39 b	2.04 a	0.26 b	0.22 b
*Sphingomonas*	1.06 bc	1.70 a	1.41 ab	1.00 c	1.23 bc
*RB41*	1.09 b	0.67 c	0.64 c	0.72 bc	1.66 a
*Candidatus*	1.42 a	1.50 a	1.48 a	0.43 b	0.75 b
*Rhizomicrobium*	1.31 a	1.10 a	1.40 a	0.24 c	0.67 b
*Pedobacter*	0.33 a	0.39 a	0.37 a	1.24 b	0.39 a
*Bacteroides*	0.99 a	0.61 a	0.23 b	0.01 c	0.01 c

Different lower-case letters indicate significant differences (*p* < 0.05) among the 5 treatments.

**Figure 3:**
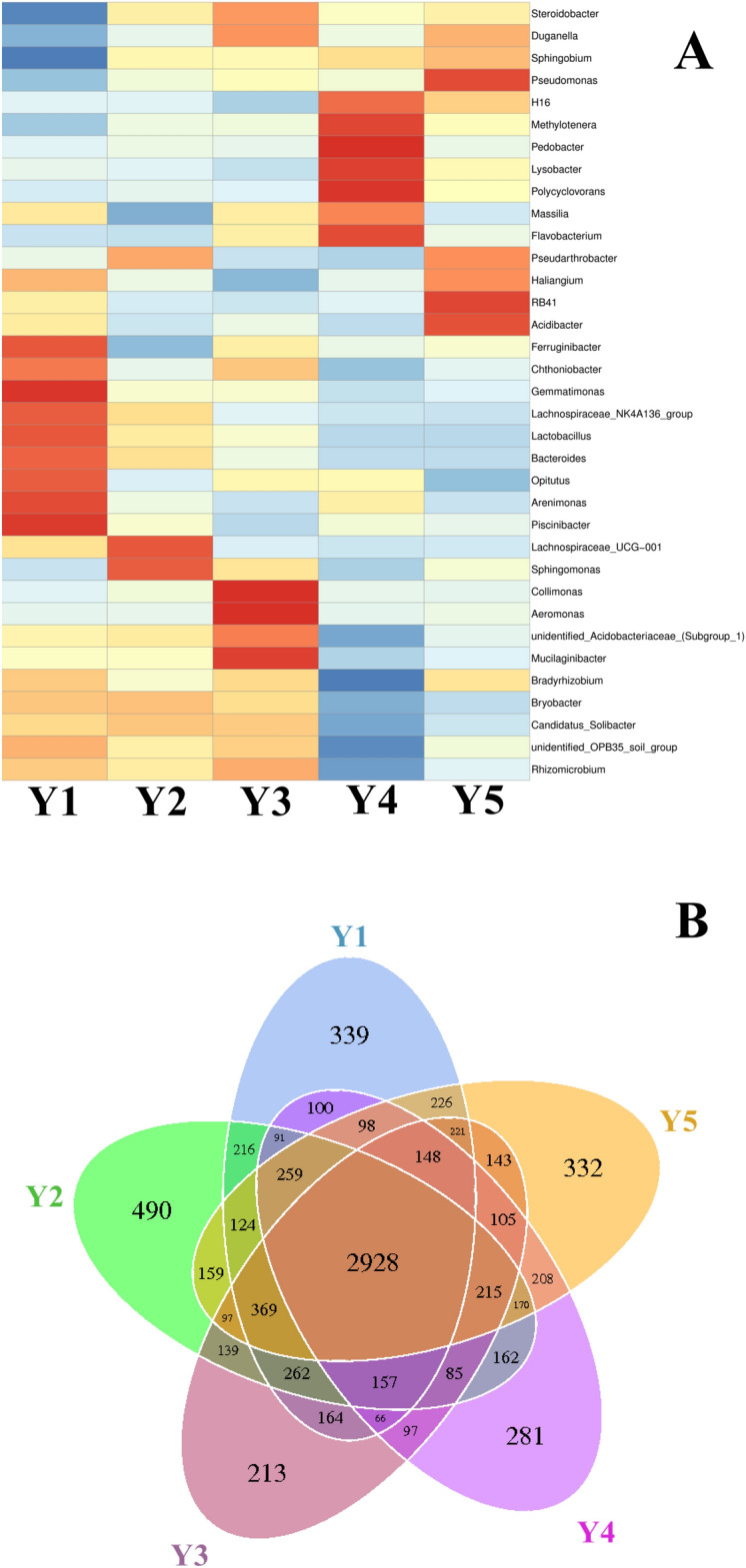
(**A**) Heat map showing the relative abundance of the top 35 most abundant bacterial genera in the samples over years and (**B**) Venn diagram of the OTUs in the samples over years at 97% nucleotide sequence identity similarity, showing the common and specific OTU numbers in the samples.

The heat map of the bacterial genera with the top 35 relative abundances in each sample showed a significant difference between the dominate bacterial communities in the *F. taipaiensis* rhizospheric soils with different cuntivation years (Fig. 3A). The dominant bacterial genera in *F. taipaiensis* rhizospheric soil with different cultivation years were Y1: *Haliangium*, *Ferruginibacter*, *Chthoniobacter*, *Germmatimonas*, *Bacteroides*, *Lactobacillus*, *Opitutus*, *Arenimonas* and *Piscinibacter*; Y2: *Pseudarthrobacter*, Lachnospiraceae_UCG_001 and *Sphingomonas*; Y3: *Steroidobacter*, *Duganella*, *Collimonas*, *Aeromonas*, unidentified Acidobacteriaceae_(Subgroup_1) and *Mucilaginibacter*; Y4: H16, *Methylotenera*, *Pedobacter*, *Lysobacter*, *Polycyclovorans*, *Massilia* and *Flavobacterium*; Y5: *Duganella*, *Pseudomonas*, *Pseudarthrobacter*, *Haliangium*, RB41 and *Acidibacter*. As shown in the Venn diagram, the samples with different cultivation years shared 2 928 common OTUs, the number of the unique OTUs in each sample from Y1 to Y5 was 339, 490, 213, 281 and 332, respectively (Fig. 3B). It indicated that the sample Y2 was greatly different with the other samples in bacterial community structures.

Heat map was created via QIIME (V1.9.1, http://qiime.org/scripts/split_libraries_fastq.html). The values corresponding to the heat map are Z-values obtained by standardizing the relative abundances of the species in each row. The Z-value of a sample in a certain classification means the difference between the relative abundance of the sample and the average relative abundance of all samples divided by the standard deviation of all samples in the classification.

### NMDS and adonis analysis

**Figure 4:**
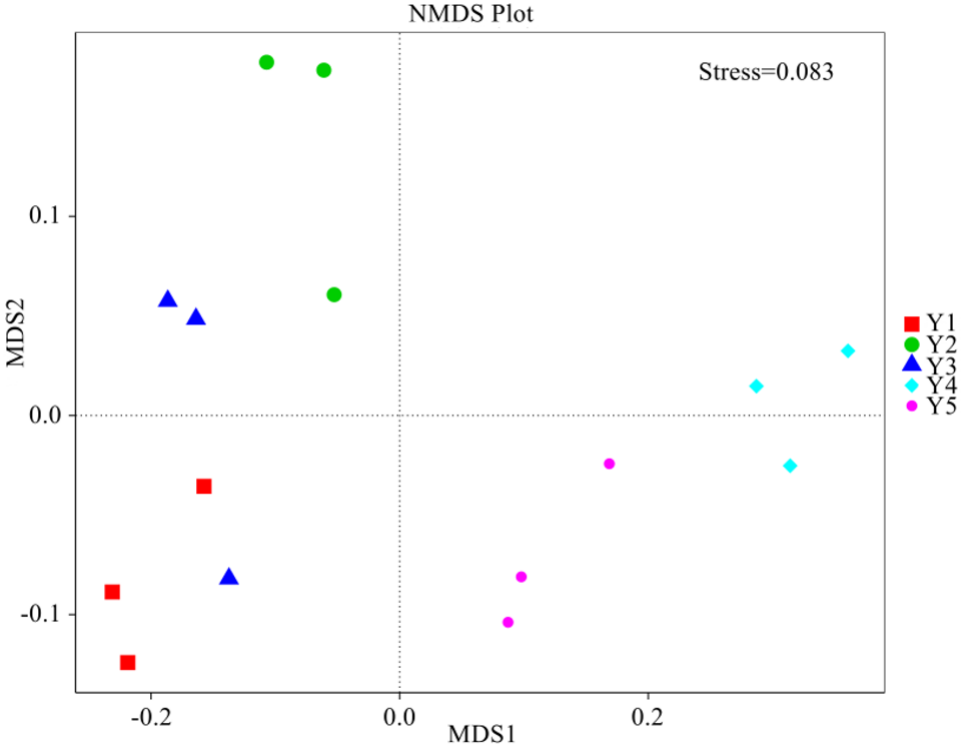
Non-Metric Multi-Dimensional Scaling (NMDS) analysis. The differences between the samples or the groups were expressed by the distance in figure, and the nearer distance showed the more similar bacterial community structure between the two samples. NMDS can accurately reflect the difference between samples when Stress < 0.2.

As shown in Fig. 4, the points representing samples Y1 and Y3 overlapped in their distribution ranges to a certain degree. However, the points representing these two samples were far from the other samples, indicating the rhizospheric bacterial communities in Y1 and Y3 were similar but were significantly different from the others. The above result was similar to that of UPGMA clustering. The further analysis by Adonis showed there were significant differences in rhizospheric bacterial communities between Y1 and Y2, Y2 and Y3, Y1 and Y5, Y3 and Y5 (*p* = 0.001) (Table 4).

**Table 4:** Adonis analysis between groups.

Vs Groups	Df	Sums of Sqs	Mean Sqs	F. Model	R^2^	*p*
Y1/Y2	1	0.128	0.128	3.454	0.463	0.001
Y1/Y3	1	0.157	0.157	4.589	0.534	0.100
Y1/Y4	1	0.479	0.479	15.058	0.790	0.100
Y1/Y5	1	0.279	0.279	7.781	0.660	0.001
Y2/Y3	1	0.079	0.079	2.557	0.390	0.001
Y2/Y4	1	0.324	0.324	11.478	0.742	0.100
Y2/Y5	1	0.192	0.192	5.941	0.598	0.100
Y3/Y4	1	0.358	0.358	14.044	0.778	0.100
Y3/Y5	1	0.212	0.212	7.208	0.643	0.001
Y4/Y5	1	0.142	0.142	5.255	0.568	0.100

Df: Degree of freedom; Sums of Sqs: Total variance; Mean Sqs: Mean square, Sums of Sqs/Df; F. Model: F inspection value ; R^2^: The explanation degree of the differences among different groups of samples, i.e. the ratio of grouping variance to total variance, the larger R^2^ means the higher the explanation of the difference; *p*: *p* < 0.05 indicates that the reliability of the test is high.

### Relationship between the rhizospheric bacterial community and the soil bio-chemical factors

The correlation analysis of the soil bio-chemical factors showed: the pH was significantly negatively correlated with the contents of available phosphorus, available nitrogen, organic matter, and the activity of urease (*p* < 0.01), was significantly positively correlated with the activity of invertase (*p* < 0.01), and was negatively correlated with the activity of alkaline phosphatase (*p* < 0.05); the activity of acid phosphatase was significantly positively correlated with the content of available potassium (*p* < 0.01); the activity of alkaline phosphatase was significantly positively correlated with the contents of available phosphorus, available nitrogen, organic matter and the activity of urease (*p* < 0.01), and was significantly negatively correlated with the activities of protease and invertase (*p* < 0.01); the urease activity was significantly positively correlated with the contents of available phosphorus, available nitrogen and organic matter (*p* < 0.01), was significantly negatively correlated with the activity of invertase (*p* < 0.01), and was negatively correlated with the protease activity (*p* < 0.05); the activity of invertase was significantly negatively correlated with the contents of available phosphorus, available nitrogen, organic matter, and was significantly positively correlated with the activity of protease (*p* < 0.01); the activity of protease was significantly negatively correlated with the contents of available phosphorus and available nitrogen (*p* < 0.01), and was negatively correlated with the content of organic matter (*p* < 0.05); the activity of catalase was positively correlated with the content of organic matter (*p* < 0.05); the content of organic matter was significantly positively correlated with the content of available phosphorus (*p* < 0.01), and was positively correlated with the content of available nitrogen (*p* < 0.05); the content of available nitrogen was significantly positively correlated with the content of available phosphorus (*p* < 0.01) (Table 5).

**Table 5:** Correlation analysis of the bio-chemical factors in the rhizospheric soil of *F. taipaiensis* with different cultivation years.

	pH	Acid phosphatase	Alkaline phosphatase	Urease	Invertase	Protease	Catalase	Organic matter	Available nitrogen	Available phosphorus
Available potassium	− 0.429	0.870**	0.372	0.184	0.135	0.134	0.522	0.592	− 0.087	0.528
Available phosphorus	− 0.762**	0.381	0.936**	0.796**	− 0.754**	− 0.748**	0.417	0.945**	0.702**	
Available nitrogen	− 0.747**	0.089	0.709**	0.825**	− 0.889**	− 0.796**	− 0.131	0.630*		
Organic matter	− 0.695**	0.498	0.874**	0.731**	− 0.645**	− 0.636*	0.521*			
Catalase	− 0.091	0.504	0.362	− 0.006	0.073	− 0.043				
Protease	0.491	0.194	− 0.806**	− 0.597*	0.940**					
Invertase	0.643**	0.173	− 0.766**	− 0.683**						
Urease	− 0.806**	0.458	0.764**							
Alkaline phosphatase	− 0.568*	0.242								
Acid phosphatase	− 0.487									

*p < 0.05, **p < 0.01.

Another correlation was established between the soil bio-chemical factors and the OTU numbers of the bacterial genera with the top 15 relative abundances: the pH was significantly positively correlated with the abundances of *Methylotenera*, *Ralstonia* and *Flavobacterium* (*p* < 0.01), was positively correlated with the abundance of *Pedobacter* (*p* < 0.05), was significantly negatively correlated with the abundances of *Bryobacter*, *Candidatus* and *Rhizomicrobium* (*p* < 0.01), and was negatively correlated with the abundances of *Lactobacillus* and *Gemmatimonas* (*p* < 0.05); the acid phosphatase activity was significantly negatively correlated with the abundance of *Flavobacterium* (*p* < 0.01), negatively correlated with the abundances of *Methylotenera* and *Collimonas* (*p* < 0.05), and was positively correlated with the abundances of *Gemmatimonas* and RB41 (*p* < 0.05); the alkaline phosphatase activity was significantly positively correlated with the abundances of *Lactobacillus*, *Gemmatimonas* and *Bryobacter* (*p* < 0.01), was significantly negatively correlated with the abundances of *Pseudomonas* and *Sphingobium* (*p* < 0.01), was positively correlated with the abundances of *Candidatus*, *Rhizomicrobium* and *Bacteroides* (*p* < 0.05), and was negatively correlated with the abundances of *Methylotenera* and *Ralstonia* (*p* < 0.05); the urease activity was significantly positively correlated with the abundances of *Lactobacillus*, *Bryobacter*, *Candidatus* and *Rhizomicrobium* (*p* < 0.01), was positively correlated with the abundance of *Gemmatimonas* and *Bacteroides* (*p* < 0.05), was significantly negatively with the abundances of *Methylotenera* and *Flavobacterium* (*p* < 0.01), and was negatively correlated with the abundance of *Ralstonia* (*p* < 0.05); the invertase was significantly positively correlated with the abundances of *Ralstonia* and RB41 (*p* < 0.01), was positively correlated with the abundances of *Methylotenera* and *Pseudomonas* (*p* < 0.05), was significantly negatively correlated with the abundances of *Bryobacter*, *Candidatus* and *Rhizomicrobium* (*p* < 0.01), and was negatively correlated with the abundance of *Lactobacillus* (*p* < 0.05); the protease activity was significantly positively correlated with the abundances of *Pseudomonas* and RB41 (*p* < 0.01), was positively correlated with the abundance of *Ralstonia* and *Collimonas* (*p* < 0.05), was significantly negatively correlated with the abundance of *Bryobacter* (*p* < 0.01), and was negatively correlated with the abundances of *Lactobacillus*, *Candidatus* and *Rhizomicrobium* (*p* < 0.05); the catalase activity was positively correlated with the abundance of *Gemmatimonas* and was negatively correlated with the abundance of *Sphingomonas* (*p* < 0.05); the available nitrogen content was significantly positively correlated with the abundances of *Bryobacter*, *Candidatus* and *Rhizomicrobium* (*p* < 0.01), was positively correlated with the abundance of *Lactobacillus* and *Sphingomonas* (*p* < 0.05), and was negatively correlated with the abundances of *Methylotenera*, *Ralstonia* and *Flavobacterium* (*p* < 0.05); the available phosphorus content was significantly positively correlated with the abundances of *Lactobacillus*, *Gemmatimonas*, *Bryobacter*, *Candidatus* and *Rhizomicrobium* (*p* < 0.01), was positively correlated with the abundance of *Bacteroides* (*p* < 0.05), was significantly negatively correlated with the abundances of *Methylotenera*, *Ralstonia* and *Sphingobium* (*p* < 0.01), and was negatively correlated with the abundances of *Pseudomonas* and *Flavobacterium* (*p* < 0.05); the content of organic matter was significantly positively correlated with the abundances of *Lactobacillus* and *Gemmatimonas* (*p* < 0.01), was positively correlated with the abundances of *Bryobacter*, *Candidatus* and *Rhizomicrobium* (*p* < 0.05), was significantly negatively correlated with the abundances of *Methylotenera*, *Pseudomonas*, *Ralstonia* and *Sphingobium* (*p* < 0.01), and was negatively correlated with the abundance of *Flavobacterium* (*p* < 0.05) (Table 6).

**Table 6:** Correlation analysis of the bio-chemical factors and the dominant genera in the rhizospheric soil of *F. taipaiensis* with different cultivation years.

	pH	Acid phosphatase	Alkaline phosphatase	Urease	Invertase	Protease	Catalase	Available nitrogen	Available phosphorus	Available potassium	Organic matter
*Methylotenera*	0.943**	− 0.631*	− 0.611*	− 0.746**	0.546*	0.419	− 0.305	− 0.618*	− 0.816**	− 0.631	− 0.825**
*Pseudomonas*	0.142	0.015	− 0.729**	− 0.324	0.952*	0.677**	− 0.327	− 0.439	− 0.620*	− 0.092	− 0.688**
*Lactobacillus*	− 0.580*	0.348	0.727**	0.776**	− 0.523*	− 0.520*	0.060	0.548*	0.781**	0.574	0.741**
*Gemmatimonas*	− 0.619*	0.588*	0.819**	0.598*	− 0.437	− 0.480	0.631*	0.438	0.885**	0.784	0.884**
*Ralstonia*	0.700**	− 0.103	− 0.569*	− 0.541*	0.711**	0.553*	0.054	− 0.637*	− 0.666**	− 0.197	− 0.658**
*Bryobacter*	− 0.884**	0.176	0.643**	0.803**	− 0.806**	− 0.692**	− 0.052	0.853**	0.752**	0.162	0.626*
*Sphingobium*	0.343	− 0.398	− 0.691**	− 0.463	0.435	0.500	− 0.512	− 0.336	− 0.726**	− 0.624	− 0.841**
*Flavobacterium*	0.810**	− 0.797**	− 0.415	− 0.737**	0.275	0.138	− 0.234	− 0.583*	− 0.567*	− 0.555	− 0.640*
*Collimonas*	− 0.291	− 0.533*	0.011	0.035	− 0.480	0.557*	− 0.408	0.249	0.115	− 0.314	− 0.101
*Sphingomonas*	− 0.427	− 0.032	0.005	0.453	− 0.328	− 0.217	− 0.588*	0.548*	0.004	− 0.444	− 0.155
RB41	0.036	0.598*	− 0.476	− 0.180	0.723**	0.766**	0.120	− 0.499	− 0.321	0.345	− 0.242
*Candidatus*	− 0.837**	0.167	0.551*	0.806**	− 0.727**	− 0.604*	− 0.205	0.760**	0.664**	0.165	0.520*
*Rhizomicrobium*	− 0.849**	0.208	0.558*	0.768**	− 0.696**	− 0.543*	− 0.134	0.681**	0.700**	0.310	0.559*
*Pedobacter*	0.518*	− 0.308	− 0.245	− 0.401	0.241	− 0.048	− 0.029	− 0.337	− 0.346	− 0.518	− 0.414
*Bacteroides*	− 0.478	0.316	0.597*	0.527*	− 0.420	− 0.449	0.173	0.491	0.623*	0.472	0.667

*p < 0.05, **p < 0.01.

**Figure 5:**
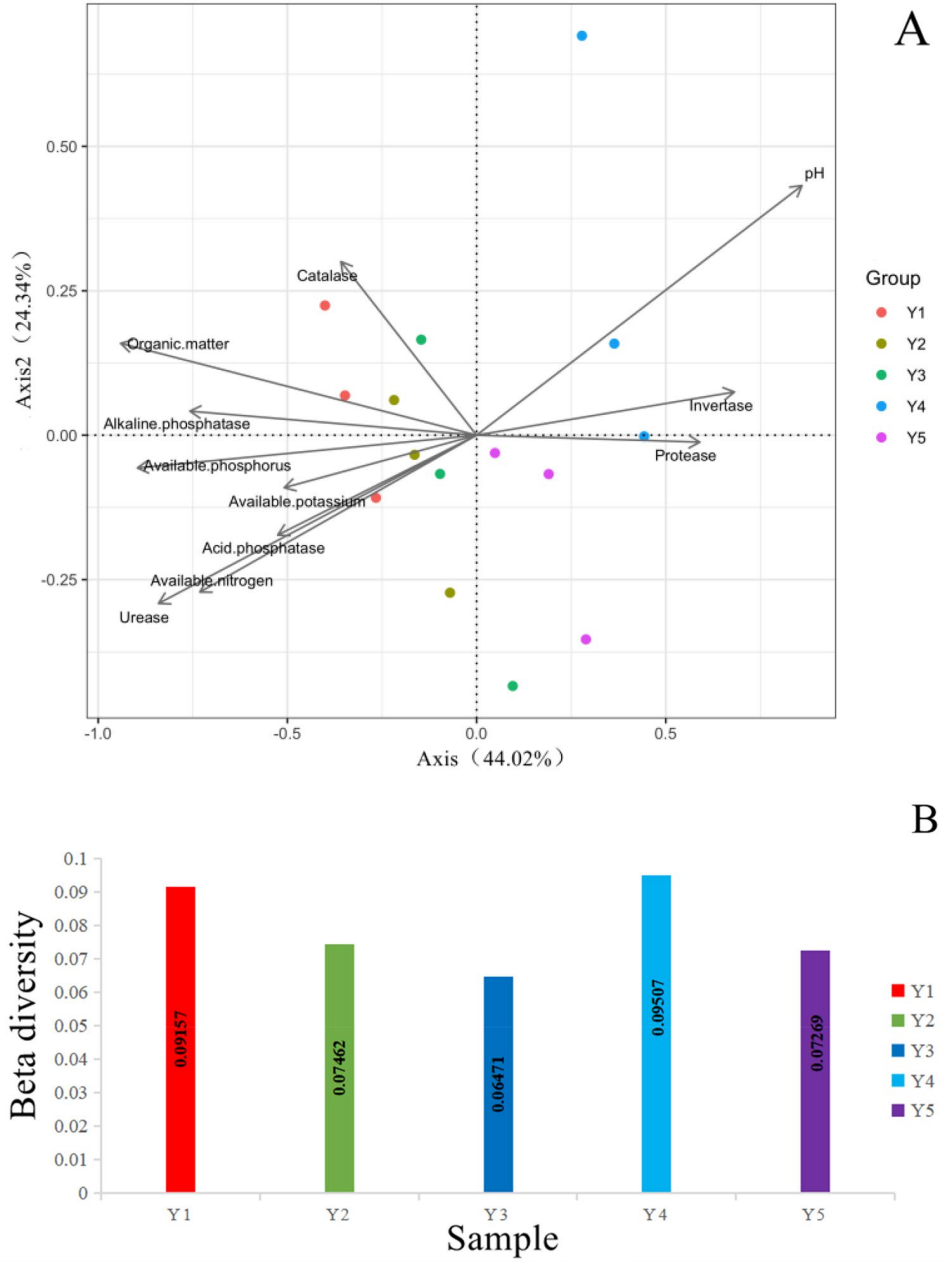
(**A**) db-RDA on bacterial communities and soil bio-chemical factors and (**B**) Intra group beta diversity bacterial communities in the rhizospheric soils of *F. taipaienses* with different cultivation years. Acid phosphatase: activity of acid phosphatase; Alkaline phosphatase: activity of alkaline phosphatase; Catalase: activity of catalase; Invertase: activity of invertase; Organic matter: content of organic matter; Protease: activity of protease; Urease: activity of urease; Available nitrogen: content of available nitrogen; Available phosphorus: content of available phosphorus; Available potassium: content of available potassium.

The results of the db-RDA showed the effect of the bacterial community on the soil bio-chemical factors in the *F. taipaiensis* rhizospheric soils with different cultivation years explained 68.36% of the total characteristic value (Fig. 5 A). Further analysis showed that the pH (R^2^ = 0.9005, *p* = 0.001), the urease activity (R^2^ = 0.7792, *p* = 0.001), the available phosphorus content (R^2^ = 0.7890, *p* = 0.001) and the organic matter content (R^2^ = 0.8564, *p* = 0.001) were the dominate factors that affected by the change of the rhizospheric soil bacterial community structure, among which the pH was the most significant factor (Supplementary Table S1). The average distance from the point to the center in each group of the PCoA ranking result based on the Bray–curtis distance matrix could represent the beta diversity within group. And the beta diversity of the bacterial community within group showed an upward trend after an initial decrease with the cultivation year (Fig. 5 B).

## Discussion

The Shannon and Simpson indices are indicators reflecting the species diversity influenced by both the richness and the evenness. In this study, these two indices showed a downward trend with the increase of cultivation year which represented a decline in species diversity in the rhizospheric soil of *F. taipainensis*. While the Chao1 and ACE indices which reflect the species richness had no significant change with the cultivation years, the decline in bacterial species diversity could be reflected in the species evenness. Previous study has shown that agricultural planting could influence the relative abundances of some specific bacterial communities in the soil which obviously alter the evenness of the bacterial communities in the soil, while the bacterial community richness would be less affected^20^. Another study have shown that plants can shape their rhizosperic microbiome structure by the root exudates during the growth, and then directionally change the rhizospheric microbial community structure, as a result, the microbial community structures in the planting soils gradually converged^28^. The downward trend showed by the intra group beta diversity of the bacterial communities from sample Y1 to sample Y4 could confirm this phenomenon. The results of UPGMA, NMDS and Adonis showed that the bacterial community structures in the soils with the adjacent cultivation years were closer to each other in clustering, which indicated that the bacterial community structure in *F. taipaiensis* rhizospheric soil changed gradually rather than suddenly with the cultivation year.

Long-term single planting is considered as a process of pathogen accumulation and soil nutrient status deterioration^26^. The dominant bacterial genera in Y1 were mainly the species that could degrade organic carbon to promote the organic cycling in soil or participate in soil nutrient cycling and the species could produce secondary metabolites to protect plants from diseases or promote the growth of plants: *Haliangium* produces haliangicin that can inhibit the growth of a wide spectrum of fungal pathogen^29^; *Ferruginibacter* and *Chthoniobacter* play important roles in soil carbon mineralization and carbohydrate metabolism^30,31^; species in genus *Piscinibacter* are methylotrophic bacteria that participate in the soil carbon cycle to increase organic matter in soil^32^; *Germmatimonas* is one of the most dominant groups in agricultural ecosystems that take part in nitrogen metabolism^33^; *Opitutus* is strongly positive correlate with NO^3−^-N in the soil^34^; *Arenimonas* species have catalytic activities of phosphatase and multiple lipases^35^; *Bacteroides* has the capability to produce acid that can dissolve minerals and provide nutrients for plants^36^. While with the increase of cultivation year, the bacterial species in rhizosphere of *F. taipaiensis* then gradually reflected the pathogen accumulation and soil nutrient status deterioration, for Y2 and Y3: *Pseudarthrobacter*, *Sphingomonas* and *Steroidobacter* are significantly positively correlated to the disease incidence of continuous cropping^37^, Acidobacteriaceae_(Subgroup_1) is negatively correlated to the content of soil organic carbon^38^; for Y4 and Y5: *Flavobacterium*, *Duganella* and *Pseudomonas* are pathogen suppressive, and are more abundant in fungal pathogen diseased plant rhizosphere^39^, the abundance of *Acidibacter* is positively correlated with the damage of plant cell^40^.

Previous studies have confirmed that pH is one of the most important factors affecting the soil bacterial community structure^41–44^. Here, our study proves that pH is also the most significant factor affected by the soil bacterial community structure and its explanation degree exceeds 90%. However, previous studies have shown that, with the increase of continuous cropping years, the pH in the planting soil would gradually decrease, resulting in soil acidification^41–46^. Therefore, the increase of the pH in the rhizosphere of *F. taipaiensis* with the cultivation year in this study provided a special case. Among the genera with the top 15 relative abundances, *Flavobacterium* and *Ralstonia* were significantly positively correlated with pH while *Bryobacter*, *Candidatus* and *Lactobacillus* were negatively correlated with pH in this study. *Flavobacterium* is a kind of important soil saprophytic bacteria, the ammonia produced by the process of its organic matter degradation can alkalize the soil^47^, the increasing trend of pH from Y1 to Y4 may also associated to the increasing of *Flavobacterium*. In addition, the ammonia produced by the process of *Flavobacterium*’s organic matter degradation may be toxic to *F. taipaiensis*. The suitable soil environment for the growth of *Fritillaria* plants is weakly acidic (pH = 6.66)^48^, therefore, in the actual planting, the rising soil pH may cause the growth and quality decline of *F. taipaiensis*. The metabolic process of *Ralstonia* can produce alkaline substances that could rise the soil pH^49^. The relative abundance of *Ralstonia* in sample Y4 was obviously higher than those in Y1, Y2 and Y3, and The pH of Y4 was 7.47, the weak alkaline environment can also associated to the increasing of *Ralstonia*, which can badly affect the growth of *F. taipaiensis*. *Bryobacter*, *Candidatus* and RB41 are affiliated to Acidobacteria, the abundances of bacteria in Acidobacteria are generally negatively correlated with soil pH^50,51^, the decline on the relative abundance of these 3 genera may also lead the soil pH rising. In addition, the relative abundance of *Lactobacillus* which can produce lactic acid, gradually declined to 0% in Y4 and Y5, that process hastened the rising of soil pH.

Some studies found that with the cultivation years’ increase, the abundances of bacteria in the cultivated soil would decline while the abundance of fungi would rise, the soil microbial population type would turn to fungal type from bacterial type, and the fungal plant pathogens would accumulate in soil during this process^52–54^. Previous studies showed that when the relative abundance of plant pathogenic fungi *Fusarium* rose, the the growth of *Gemmatimonas* could be seriously inhibited, and the relative abundance of *Pseudomonas* would rise^55^. Therefore, in this study, the decline of the *Gemmatimonas*’s relative abundance and the increase of the *Pseudomonas*’s relative abundance may corresponded to the accumulation of pathogenic fungi with the cultivation years. In addition, *Gemmatimonas* was positively correlated with the activity of phosphatase and the contents of available phosphorus and organic matter in this study. Previous study have found that *Gemmatimonas* can dissolve insolubilized phosphate and convert it into solubilized phosphate for plant growth^56^, its decline in relative abundance would hasten the impoverishment of planting soil. Moreover, the bacteria that declined in the relative abundance include various kind of nitrogen fixing bacteria, potash dissolving bacteria and phosphate dissolving bacteria^53^. Thus, the above conclusion explained the reasons for the decline of the contents of available nitrogen, available phosphorus and available potassium.

Urease and protease are the key enzymes involved in the transformation of soil organic nitrogen to inorganic nitrogen, which play an extremely important role in the process of soil nitrogen cycling^57^. Urease can promote the hydrolysis of urea to ammonia in soil, its activity is often used to characterize the intensity of soil nitrogen transformation^58^. Protease can promote the hydrolysis of proteins and peptides to amino acids, and its activity is an important characterization of soil nitrogen supply capacity^59^. The significant higher protease activities in Y4 and Y5 compared with those in Y1, Y2, and Y3 may corresponded to the increase of *Collimonas* which was positively correlated with protease activities in Y3, for the *Collimonas*’s metabolites had high protease activity^54^. The changing trend of urease activity was opposite to that of protease and the urease activity was also negatively correlated with the protease activity in this study, which was consist with the result of Chang et al^60^. Previous study found that high protease activity in the soil could significantly increase soil total nitrogen content, while the excessive total nitrogen content could inhibit the secretion of urease^61^. Another study showed that the soil urease activity was positively correlated with the organic matter content and was negatively correlated with the invertase activity^62^, which was consist with our study. Therefore, in our study, the urease activity showed a changing trend similar with the organic matter content and contrary to the invertase activity.

Moreover, in the actual production, the cultivation of *F. taipaiensis* was mainly began as seed sowing, which usually took at least 5 years from germination to harvest^63^. And because of this long-term single planting pattern, the plant would release large amount of secondary metabolites such as phenolic acids into the soil which would be toxic to the plants and affect the soil microbial structure^64–67^. Study have confirmed that phenolic acids could reduce the number of beneficial microbial population under the condition of long-term single planting^68^. Previous study on *F. taipaiensis* also found that the number of culturable beneficial bacteria in the rhizospheric soil would significantly decline in the 3rd and 4th cultivation years during the single planting^9^, this phenomenon could be explained by the accumulation of phenolic acids that also badly affect the growth of *F. taipaiensis.*

## Conclusion

In summary, long-term single planting pattern cause the obvious change of the bacterial community structure in the *F. taipaiensis* rhizospheric soil, the evenness of the soil bacterial community declined, the relative abundance of the beneficial bacteria decreased and the pathogenic microorganisms accumulated. The decrease of the available nitrogen, available phosphorus and organic matters impoverished the soil. This study can provide a preliminary scientific basis for the soil management in the sustainable cultivation of *F. taipaiensis*, solving the germplasm decline problem appeared in the production of *F. taipaiensis* by combining with reasonable rotation, application of organic fertilizer, development of biological fertilizer and control of harmful pathogens. In our future research, we are going to use metagenomic analysis to identify more enzymes and proteins that may be involved in the changes on the bio-chemical properties of *F. taipaiensis* rhizospheric soil, moreover, we will analyze the relationship between the rhizospheric microorganisms and the growth and the medicinal quality of *F. taipaiensis* to find the potentially beneficial microorganisms that can be used in the sustainable cultivation of *F. taipaiensis*.

## Supplementary Information

Supplementary Information.

## Data Availability

All obtained sequences data and the raw data of the analysis can be downloaded at https://ngdc.cncb.ac.cn/gsa/browse/CRA007387.
