# Supplementary material for: The rhizospheric bacterial diversity of Fritillaria taipaiensis under single planting pattern over five years
Source: Sci Rep. 2022 Dec 29;12:22544. doi: 10.1038/s41598-022-26810-x (PMC9800406; doi:10.1038/s41598-022-26810-x)
Supplement: Supplementary file 1 — Supplementary Information. [file 41598_2022_26810_MOESM1_ESM.docx]

**Table S1. Redundancy analysis of the effect of the bacterial community on the soil bio-chemical factors**

| Soil factor | Axis1 | Axis2 | R^2^ | *p* |
| --- | --- | --- | --- | --- |
| pH | 0.92138 | 0.38866 | 0.9005 | 0.001 *** |
| Protease | 0.99849 | 0.05485 | 0.3370 | 0.079 |
| Catalase | -0.86655 | 0.49909 | 0.1742 | 0.260 |
| Urease | -0.95376 | -0.30057 | 0.7792 | 0.001 *** |
| Acid phosphatase | -0.95705 | -0.28994 | 0.3015 | 0.116 |
| Alkaline phosphatase | -0.99956 | -0.02968 | 0.5541 | 0.008 ** |
| Invertase | 0.98911 | 0.14715 | 0.4617 | 0.017 * |
| Available nitrogen | -0.94900 | -0.31528 | 0.5985 | 0.002 ** |
| Available phosphorus | -0.99340 | -0.11466 | 0.7890 | 0.001 *** |
| Available potassium | -0.98099 | -0.19405 | 0.2630 | 0.159 |
| Organic matter | -0.99853 | 0.05425 | 0.8564 | 0.001 *** |

R^2^ is the characteristic value of different soil factors on bacterial community structure, "***": *p*＜0.001, "**": *p*＜0.01, "*": *p*＜0.05.
